# A flexible computer-to-computer protocol for DISNET: a Distributed Instrument System NETwork

**DOI:** 10.1155/S1463924684000377

**Published:** 1984

**Authors:** Paul J. Gemperline, Robert Megargle

**Affiliations:** Department of Chemistry Cleveland State University Cleveland Ohio 44115 USA


Journal of Automatic Chemistry, Volume 6, Number 4 (October–December 1984), pages 192–196

### A flexible computer-to-computer protocol for DISNET: a Distributed Instrument System NETwork

**Paul J. Gemperline and Robert Megargle**

*Department of Chemistry, Cleveland State University, Cleveland, Ohio 44115, USA*

### Introduction

Several different laboratory networks have been described in the chemical literature [1–14]. Nearly all of them were based on a hierarchical scheme where one or more time-share host computers provided communication services to remote satellite computers. All used a star topology. The satellites performed real-time data acquisition for instruments. Communication was most frequently implemented via interactive commands available through the time-shared system. Network operation was limited to host-satellite communications in all but two cases [8 and 13]. Typical applications included down-loading of programs, remote storage of data, and batch processing of print or plot files.

The advantages offered by these hierarchical systems were: (1) easy implementation of communication protocols by use of interactive time-share commands; and (2) readily available host software for applications. All of the hierarchical networks share the disadvantage of being wholly dependent on the central time-share host. Hardware problems in the host can disable all satellites and communication is usually restricted between hosts and satellites. Satellite-to-satellite communication would allow for easier expansion of the network. Finally, changes and new versions of the time-shared host may necessitate modification of every satellite, making replacement of the host impractical unless the new system is largely compatible with the old system. While the initial development of DISNET [15] required more effort, its flexibility will easily accomodate new systems. In the long run, this advantage offsets the effort required for implementation.

The recent availability of commercial general-purpose networks has greatly reduced the development effort required to construct a laboratory network. Some have shortcomings for laboratory applications, but some are quite suitable.

### The DISNET communication protocol

A network protocol is a set of rules for orderly communication which guarantees message integrity and avoids the chaos of *ad hoc* communication. Unique protocols have been developed for nearly every type of network hardware constructed. In 1981 the International Standards Organization issued a standard for *Open Systems Interconnection*, which established guidelines for developing protocols. Seven layers of control are identified, each building on the previous layer to provide increasingly sophisticated control as well as a higher level of abstraction. The DISNET protocol has been developed with these objectives in mind.

In the DISNET system, a station which initiates a communication becomes a 'control station'. Part of the transmission line acquisition sequence is to send the address of the station with which it wishes to communicate, which becomes the 'slave station'. All activity that occurs from the time a station gains control of DISNET until DISNET is again idle is called a 'transaction'. The lowest level of software, the Primitive Device Handler (PDH), combines the transmission of individual data bytes to construct transactions. Heading bytes and suffix bytes are added to provide message routing and error-detection information.

A series of related transactions between the same pair of stations is called a 'session'. Transactions within a session can be initiated by either station, allowing data to be transmitted in both directions. Many transactions can take place during the course of a session and the transmission hardware is often idle for extended periods of time between transactions. It is free for use by other application programs during these times, allowing many sessions to proceed concurrently. Sessions are controlled by the second layer of software: the DISNET Channel Control Module (CCM).

The CCM manages eight channels at each station. Each channel is associated with a session. Channel zero is used for supervisory functions only and the other seven are available for general use. Sessions are started and stopped by assigning and releasing channels through supervisory messages that are sent over channel zero. Once channels are open, the send and receive data supervisor calls allow partners to initiate or accept transactions. The CCM routes transactions to the proper destination, ensures message integrity, and prevents unauthorized communication from third parties. Data integrity is ensured through automatic retransmission of transactions found to be in error.

Application programs are the highest level of software. At this level, programmers have the freedom to design suitable types of communication protocols using the existing channel supervisor calls, although a standard protocol has been defined to achieve resource sharing. The protocol makes a distinction between two types of communicating partners, resource providers and resource consumers. Making this distinction helps the designer address communication problems in a straightforward manner.

---

Paul Gemperline is now based at the Department of Chemistry, East Carolina University, Greenville, North Carolina 27834, USA. Correspondence should be addressed to Robert Megargle.

### DISNET Primitive Device Handler

The Primitive Device Handler (PDH) performs hardware manipulations in response to DISNET interrupts. It uses data areas created by the CCM to construct heading information for transactions. When transactions are received at a slave, the heading information is checked for errors. Data to be transmitted is placed in buffers by the sending application program and given to the PDH. Received data is packed by the PDH into buffers supplied by the receiving application program. An error-detection suffix is added by the sending station and checked at the receiving station. The PDH thus creates and enforces the standard protocol. All protocol errors, as well as hardware errors, are detected in this module.

### Transaction protocol

Three bits of pulse code data are transmitted and received by the DISNET hardware [15]. They indicate to receiving stations that a valid data byte is present on the data lines. Pulse code zero is predefined in hardware as an address transfer byte, and pulse code 7 indicates data is not present. The remaining pulse codes (1 to 6) are used to clock data into hardware registers at 'dumb' nodes. For computer-to-computer communication, it was decided to use the pulse codes to control transactions. They are interpreted by the PDH. Figure 1 shows the standard transaction protocol and the pulse code associated with each type of data byte. The transaction protocol has three major sections: a heading of two or three bytes, a body of user data, and an error-detection suffix.

| Data byte | | Pulse code |
|---|---|---|
| Control code | Slave station address | 0 |
| Control code | Control station address | 2 |
| Packet type (channel 0 only) | | 2 |
| Send record 1 | | 1 |
| End-of-record byte (ignored) | | 3 |
| Send record 2 | | 1 |
| Line reversal byte (ignored) | | 5 |
| Receive record 1 | | 1 |
| End-of-record byte (ignored) | | 3 |
| Receive record 2 | | 1 |
| Checksum byte | | 6 |
| Diagnostic byte | | 4 |

*Figure 1. Transaction protocol.*

### Transaction heading

Channel zero transactions always have three bytes of heading information inserted by the PDH. All other transactions require two heading bytes. The least significant five bits of the first byte are used for the address of the slave station and are required by the hardware. The most significant three bits specify the target channel number at the slave. The slave address byte is automatically transmitted by the hardware with a pulse code of zero.

The least significant five bits of the second byte have the address of the controlling station. The most significant three bits specify the originating channel. A pulse code of 2 indicates that this byte is part of the heading. The PDH uses this byte to verify that the transaction was initiated by the partner associated with an open channel. An illegal communication is indicated when data originates from the wrong channel and/or the wrong station. A third heading byte sent with pulse code 2 is included to specify the packet type for channel zero transactions and is optional for other channels.

### User data records

The control station always sends or receives at least one record following the heading. User data is sent with a pulse code of 1. The PDH uses tables provided by application programs to determine how many bytes are to be sent or received.

Three different conditions can specify the end of a send record. (1) The end-of-record byte with pulse code 3 indicates that another send record will follow. In this case, the handler sets up pointers to the next buffer. There can be many such send records in a transaction. (2) A line reversal byte with pulse code 5 indicates both the end of a send record and a line reversal. If this happens, the slave is then required to send at least one record. The value of end-of-record and line reversal bytes is immaterial. After line reversals occur, slave send records are terminated with either an end-of-record byte (another record will follow) or a checksum byte. Only one line reversal for data is legal. (3) The third possibility is receipt of a checksum byte. This indicates either the end of send records or the end of receive records, and it causes the transaction termination sequence to begin.

### Termination sequence

The checksum byte is an eight bit sum of all bytes sent and received, including heading bytes, line reversal bytes and end-of-record bytes. The last station which was sending (either slave or control) calculates the two's complement of this sum and transmits it with a pulse code of 6. The last station receiving adds this byte to its own checksum. The result should be zero. If not, a data-transfer error has been detected by the receiver.

Once the checksum byte is received, the control station reverses the line so that the station which received the checksum can send the diagnostic byte with a pulse code of 4. A diagnostic byte of zero is sent when no errors are detected during the transaction, and a value of 2 is sent when the checksum disagrees. If other errors are detected at the control station, a non-zero diagnostic byte will be sent immediately with pulse code 4. If a slave station detects an error, it must wait for the first opportunity to send the diagnostic byte since slave stations cannot reverse the direction of transmission. They discard data until the line reverses. If more than 32 K bytes are discarded, the receiver stops taking data to force the hardware to abort the apparent run-away condition. The diagnostic byte always ends a transaction, whether or not preceded by the checksum byte.

The mechanism for reversing the flow of data in the middle of a transaction is a unique feature of DISNET which is not found in most commercially available networks. It not only permits two-way data, but also allows immediate acknowledgement of transactions through the use of the diagnostic byte. In most other systems, the receiver must go through the full network acquisition sequence to transmit a separate acknowledgement to the sender. This allows DISNET to make more efficient use of the shared data pathway.

### DISNET Channel Control Module

The Channel Control Module (CCM) is the next layer of software. Supervisor or subroutine calls provide an interface for application programs. Six such calls are available, including the open channel, enable channel, close channel, reset station, send data, and receive data commands. This layer is responsible for creating and managing all eight channels, besides initiating user-requested data transfers.

### Channel Control Blocks

Application programs are required to pass up to three different types of data blocks to the CCM, depending on the function to be performed. A Channel Control Block (CCB), shown in figure 2, is always required. The first word specifies the application program that has ownership of a channel. Once assigned, other programs are not allowed to use the channel. The second word contains the channel number and is initialized when a channel is successfully opened. The third word contains two flags, the 'DONE' and 'RETRY' flags. When an operation terminates, either normally or due to errors, the DONE flag is set. The RETRY flag is set when non-fatal errors are detected. The CCM automatically retries transactions with non-fatal errors. The last word of the CCB is a code returned to the application to indicate the completion status of a function when the DONE flag is set. Table 1 lists all return codes and their meaning.

| | | | |
|---|---|---|---|
| Word 0 | Program identifier | | |
| Word 1 | Channel number* | | |
| Word 2 | DONE | Flags* | RETRY |
| Word 3 | Error code* | | |

* Initialized by the CCM.

*Figure 2. Format of a channel control block.*

A code of zero indicates the operation was completed successfully. Codes 1 to 3 are considered non-fatal errors and are automatically retried up to five times, after which they are classified as fatal errors. The hardware abort condition (error 1) usually indicates that a slave station did not respond fast enough to a DISNET interrupt. The channel busy error (error 3) is reported when a slave channel cannot receive the data being sent. This happens when there are no receive buffers at the slave. In all cases, the CCM waits for 20 ms and then retries the transaction. This allows up to 1 s for a receiver to 'catch up' or become 'free', providing wide time margins for communicating programs to synchronize their co-operative functions.

When fatal errors are encountered, the CCM initiates the printing of a message on the system console. These errors are never retried. User software errors (error 4) occur when the number or size of send records exceed the number or size of receive buffers. In this case the entire transaction is discarded. System software errors (error 5) are reported when protocol violations are detected by the PDH. A channel closed error

*Table 1. Channel return codes.*

| *Code* | *Meaning* |
|---|---|
| 0 | No error |
| 1 | Hardware abort |
| 2 | Data transfer error |
| 3 | Channel busy |
| 4 | User software error |
| 5 | System software error |
| 6 | Channel closed |
| 7 | Receiver not found |

(error 6) is reported when an application program attempts to send data through a closed channel. This code also indicates the successful completion of a 'close channel' operation, described shortly. The receiver not found error (error 7) occurs when an application program attempts to open a channel and fails to locate the target receiver.

### Channel zero transactions

Prior to opening any channels, a resource provider application program has to perform an 'enable channel' supervisor call, giving a two byte name resource name. The CCM serving the resource provider dynamically allocates a channel number to this program. Channel zero is always open and ready to accept data from CCMs in any other station. The packet heading for channel zero transactions contains an extra byte to specify the packet type. Types 1, 2, and 3 exist at the present time, and are 'open channel', 'close channel', and 'reset station' types, respectively.

*Open channel transactions*: the open channel packet is sent by the CCM when a resource consumer application program performs the open channel supervisor call. The consumer gives the address of the station where the resource provider resides and its two byte resource name. The CCM allocates a free channel to the consumer and starts the open channel transaction. During this transaction, the slave returns its list of resource names as the first receive record after a line reversal. The CCMs in both the control and slave stations independently scan this list at the end of the transaction. When a match of resource names is found, both consumer and provider application programs are informed that the channel link is open by setting the DONE flag and giving a return code of zero in the CCBs. The channel link is actually established at both CCBs by associating the independently assigned resource provider and consumer channel numbers. The two partners may now communicate directly with each other without the intervention of channel zero. If the open function fails five times in a row, the CCB return code is set to seven (requested receiver not found). The application programmer is left to decide which appropriate action must be taken in this case.

*Close channel transactions*: the second type of channel zero transaction is the close channel transaction. Either partner may initiate this transaction by use of the close channel supervisor call, giving the channel number to be closed. The CCM determines from internal lists where the partner resides and sends the close channel transaction. Once the transaction is complete, both the control and slave CCMs set a return code of six (channel closed) and the DONE flag in the CCBs to indicate the successful completion of the operation.

*Reset transaction:* the third type of channel zero transaction is reset, initiated with the reset station supervisor call. The application program specifies the address of the station to be reset. Once the reset transaction is complete, both control and slave CCMs close every channel which is open between them. Channels which are open to third parties are unaffected by this call.

### User data transactions

When an application is to act as a control for a transaction to move data between it and a slave, it must supply a 'control transaction table'. The format of this table is given in figure 3. Control transactions require that the application program send or receive at lease one record. Once an initial record has been sent, additional records can be sent or the direction of data transfer may be reversed and the slave allowed to send. The control transaction table specifies the byte count and buffer address of each record to be sent. The send records are listed sequentially. A byte count of zero is used to indicate that no more send records exist.

| | |
|---|---|
| Send record 1 | Byte count |
| | Buffer address |
| Send record *N* (optional) | Byte count |
| | Buffer address |
| Line reversal | 0 |
| Receive record 1 (optional) | Byte count |
| | Number of bytes received* |
| | Buffer address |
| Receive record *N* (optional) | Byte count |
| | Number of bytes received* |
| | Buffer address |
| End of list | 0 |

* Initialized by the CCM.

*Figure 3. Format of a control transaction table.*

The next section of the control transaction table specifies the size of a receive buffer. If the first byte count is zero, no receive buffers exist and the transaction is terminated. If non-zero, the list specifies the available size and address of each receive buffer. At the end of the transaction, the PDH saves the number of bytes actually received in each record in a third field of this table. The end of receive records (also the end of the list) is indicated when a receive buffer with the size of zero bytes is specified.

When an application program is to act as a slave, it must provide a 'slave transaction table', shown in figure 4. This list is nearly the same as a control transaction table. Since the slave is required to receive first, and send second, the order is simply reversed.

To enable the receipt of data, an application program must perform the receive data supervisor call. The CCM immediately returns to the application program, which could then poll the DONE flag to determine when the data is received. The application program can suspend itself to wait for the data. In this case, the CCM automatically restarts the application program and cancels any remaining wait time when the data is received.

If a receive buffer is not large enough to contain all of the data sent in one record, the excess data is thrown out, and a user software error (error 4) is reported. There must also be a one-to-one correspondence between send buffers and receive buffers in the control and slave stations. A user software error is reported when this condition is not met.

| | |
|---|---|
| Receive record 1 (optional) | Byte count |
| | Number of bytes received* |
| | Buffer address |
| Receive record *N* (optional) | Byte count |
| | Number of bytes received* |
| | Buffer address |
| Line reversal | 0 |
| Send record 1 | Byte count |
| | Buffer address |
| Send record *N* (optional) | Byte count |
| | Buffer address |
| End of list | 0 |

* Initialized by the CCM.

*Figure 4. Format of a slave transaction table.*

### DISNET test

A set of two programs were written to test the DISNET software system. They were designed to be used together, each as a partner in a series of transactions which exercised the system. Four different tests were performed. The first one tested the open, enable and close channel commands, followed by the send and receive data commands, including transactions with multiple records and a line reversal. After verifying that the basic commands work, the second test opened two channels simultaneously to verify that the system allowed channels to operate independently. A more sophisticated third test was then performed: four tasks, two in each station, competed for use of the communication system. Each task attempted to open or enable two channels so that four channels were in use during the middle of the test. This test exercised the mechanisms which arbitrate contention between competing tasks, as well as the automatic retry scheme which reschedules communications if they are initiated over busy channels.

The last test measured the channel burst data transfer rate, defined as the maximum rate at which data can be transferred over a channel. The overhead incurred during line acquisition and transmission of supervisor data is not included in this term, or is negligible in comparison to the amount of data sent. Once the burst rate was measured, the channel throughput data transfer rate was measured for several different size transactions. The throughput rate includes the overhead incurred during line acquisition and the added overhead of channel supervisory functions, including the occurrence of occasional retries.

The results of the DISNET tests are listed in table 2. As expected, the throughput decreases with smaller packet sizes, due to overhead time incurred while setting up the data transmission packet and waiting for the remote computer to respond. The lower than usual throughput rate for packet sizes

*Table 2. DISNET throughput data transfer rate.*

| *Packet size (bytes)* | *Data transfer rate (Kbytes/s)* |
|---|---|
| 10 | 3·704 |
| 100 | 3·012 |
| 1000 | 6·579 |
| 5000 | 7·483 |

Maximum data transfer rate = 10·167 Kbytes/s.

of 100 bytes is an artifact resulting from intermittent, automatic retransmission of data. Lengthy real-time clock interrupt service in one of the hosts sometimes caused sufficient time delays in responding to DISNET interrupts that the DISNET abort sequence was activated. The time required to transmit a 100 byte packet circumstantially coincided with the development computer's clock rate of 100 ms, thus increasing the probability of this interference when 100 packets of 100 bytes are transmitted consecutively. The automatic retransmission sequence always corrected the error but slightly lowered the data transmission rate.

Independent channel operation was also verified by this test. The multiple channel, multiple task portion of the test illustrated the ability of the CCM to successfully arbitrate contention among four active channels. Although the programs always request service for channels in the same order, some randomness is observed in the order in which requesting channels receive service. The randomness results when transactions attempt to contact busy receivers and are rescheduled by the CCM.

The system has also proven to be highly reliable in routine use. Data transmission errors have only been detected on two occasions: one was traced to a faulty integrated circuit and the other was attributed to dirty connections.

### References

1. Woodward, W. S., Ridgway, T. H. and Reilley, C. N., *Analyst*, **99** (1974), 838.
2. Woodward, W. S., and Reilley, C. N., *Pure and Applied Chemistry*, **50** (1978), 785.
3. Woodward, W. S. and Reilley, C. N., A general purpose microcomputer for laboratory automation in a hierarchial environment, In Levy, G. C. and Terpstra, D. (eds.) *Computer Networks in the Chemical Lab* (Wiley-Interscience, New York, USA, 1981).
4. Woodard, F. E., Woodward, W. S. and Reilley, C. N., *Analytical Chemistry*, **53** (1981), 1251A.
5. Kaufman, W. E. *Computers in Chemical Education and Research*, (Proceedings of the 3rd International Conference, 1976), 133. (Published in 1977.)
6. Hatfield, W. T., Goehner, R. P. and Lifshin, E. The General Electric Laboratory Automation System. In Levy, G. C. and Terpstra, D. (eds.) *Computer Networks in the Chemical Lab* (Wiley-Interscience, New York, USA, 1981), 71.
7. Dessy, R. E., *Analytica Chimica Acta*, **103** (1978), 459.
8. Dessy, R. E. and Hooley, D. J., FORTH, A fourth generation language system implements a network. In Levy, G. C. and Terpstra, D., *op. cit.*, 41.
9. Zietlow, K., Broecker, H. Ch. and Muller, H. G. W., Hardware and software aspects of the development of the satellite data processing network at the University of Hamburg. In Levy, G. C. and Terpstra, D., *op. cit.*, 161.
10. Alderman, D. W., Hamill, W. D. Jr., Mayen, C. L. and Grant, D. M., The University of Utah NMR laboratory minicomputer network. In Levy, G. C. and Terpstra, D., *op. cit.*, 178.
11. Smit, H. C., *Analytica* [illegible]*imica Acta*, **122** (1980), 207.
12. Chia-Yu Li, Barrett, T. H. Jr., Lunney, D. and Salt, A., *Analytica Chimica Acta*, **134** (1982), 167.
13. Levy, G. C., Terpstra, D. and Dumoulin, C. L., WARPATH, a computer network for a multi-user laboratory environment. In Levy, G. C. and Terpstra, D., *op. cit.*, 102.
14. Coley, R. F., A Production Laboratory Implementation of DECNET. In Levy, G. C. and Terpstra, D., *op. cit.*, 188.
15. Gemperline, P. J., Megargle, R., Dartt, A., Slivon, L. and Zadnik, V., *Journal of Automatic Chemistry*, **6** (1984), 27.

### 'FOOD ADDITIVES AND CONTAMINANTS'

Taylor & Francis launched this new journal during 1984. The editors are Drs R. Walker (University of Surrey, UK) and M. E. Knowles (Norwich, UK). Dr N. T. Crosby (Laboratory of the Government Chemist, UK) is associate editor. Forthcoming papers include:

R. C. Massey *et al.*—Total *N*-nitroso group analysis of foods. II. Further studies on the precision and sensitivity of the assay.

A. R. Tricker *et al.*—Incidence of some non-volatile *N*-nitroso compounds in cured meats.

B. Hoffmann *et al.*—Investigation on the occurrence of non-extractable residues of trienbolone acetate in cattle tissues in respect to the bioavailability and immunological reactivity.

H. H. D. Meyer *et al.*—Application of Synovex-H in veal calves: steroid release and residues.

J. C. Sherlock and G. A. Smart—Tin in foods and the diet.

P. H. Andersen and N. J. Jensen—Mutagenic investigation of flavourings: dimethyl succinate, ethyl pyruvate and aconitic acid are negative in the *Salmonella*/mammalian-microsome test.

K. Sones *et al.*—The glucosinolate content of UK vegetables—cabbage (*Brassica oleracea*), swede (*B. napus*) and turnip (*B. campestris*).

G. A. Smart and J. C. Sherlock—Tin in foods and the diet.

M. L. Bates *et al.*—Determination of synthetic growth promoters in bile.

E. H. J. M. Jansen *et al.*—A chemiluminescent immunoassay for 17 α-methyltestosterone.

J. Gilbert *et al*—Automated headspace GC-MS analysis of ethylene dibromide fumigant residues in fresh fruits.

D. H. Watson and S. M. Hones—Carcinogenic higher plant metabolites in the human diet in temperate countries.

A. L. Patey *et al.*—Ammonia caramels: specifications and analysis.

R. Walker—Sulphiting agents in foods: some risk/benefit considerations.

K. R. Price and G. R. Fenwick—Naturally occuring oestrogens in foods.

*Sample copies from the Marketing Department, Taylor & Francis Ltd, Rankine Road, Basingstoke, Hampshire RG24 0PR, UK.*

